# Human disease-relevant mutations in the VRK-1 basic-acidic-basic patch result in motor impairment independent of mitotic defects in *C. elegans*

**DOI:** 10.17912/micropub.biology.002279

**Published:** 2026-10-30

**Authors:** William Smith, Peter Meister

**Affiliations:** 1 Institute of Cell Biology, University of Bern, Bern, Switzerland

## Abstract

Mutations in Vaccinia Related Kinase 1 (VRK1) are associated with distal neuropathies in humans. Here, we report the first example of disease relevant VRK1 mutations in a model organism, and show that these cause progressive motor defects in *C. elegans* that are largely independent of mitotic issues. Our experiments suggest that this VRK-1 mutant will be a useful tool for dissecting the etiology of VRK1-related neurological disorders. In addition, we report VRK-1-positive strands that span the separating chromosome masses during anaphase in *C. elegans* embryos, and present high temporal resolution videos of VRK-1 localization during mitosis.

**Figure 1. VRK-1 4R/KA mutations result in motor but not mitotic impairment in C. elegans:** Schematic domain map of human VRK1 (hVRK1) and *C. elegans* VRK-1 (ceVRK-1), showing the kinase domain and the C-terminal intrinsically disordered region (IDR). The conserved basic-acidic-basic (BAB) patch is indicated in green, as predicted by alignment from (Budziszewski et al., 2022). **(b)** Amino acid sequence of the BAB in hVRK1 and ceVRK-1, with wildtype residues in green, and mutated residues in red. **(c)** Representative confocal images of VRK-1::wrmScarlet/HIS-72::GFP (left) or VRK-1 ^4R/KA^ ::wrmScarlet/HIS-72::GFP (right) during interphase, prophase, metaphase and anaphase in embryos. **(d)** Brood size of VRK-1::wrmScarlet (n=7) or VRK-1 ^4R/KA^ ::wrmScarlet (n=8), with each point representing the number of progeny from an individual mother. **(e)** Representative confocal images of VRK-1::wrmScarlet/HIS-72::GFP (upper) or VRK-1 ^4R/KA^ ::wrmScarlet/HIS-72::GFP (lower) in hypodermal nuclei of L3 worms. **(f)** Thrashing assay of WT VRK-1 (n=11) or VRK-1 ^4R/KA^ (n=8) in L4 larvae where each point indicates number of thrashes in 30 seconds for an individual worm. p<0.0001, Students t-test. **(g)** Thrashing assay in adult animals as described in f, at d1 (n=21, 20), d3 (n=17, 21) and d5 (n=20, 20) of adulthood for WT VRK-1::wrmScarlet or VRK-1 ^4R/KA^ wrmScarlet animals. Indicated p-values are from one-way ANOVA statistical tests comparing d1, d3 and d5 within the same genotype. **** indicates p<0.0001 when comparing VRK-1::wrmScarlet with VRK-1 ^4R/KA^ ::wrmScarlet at the same timepoint (Students t-test).

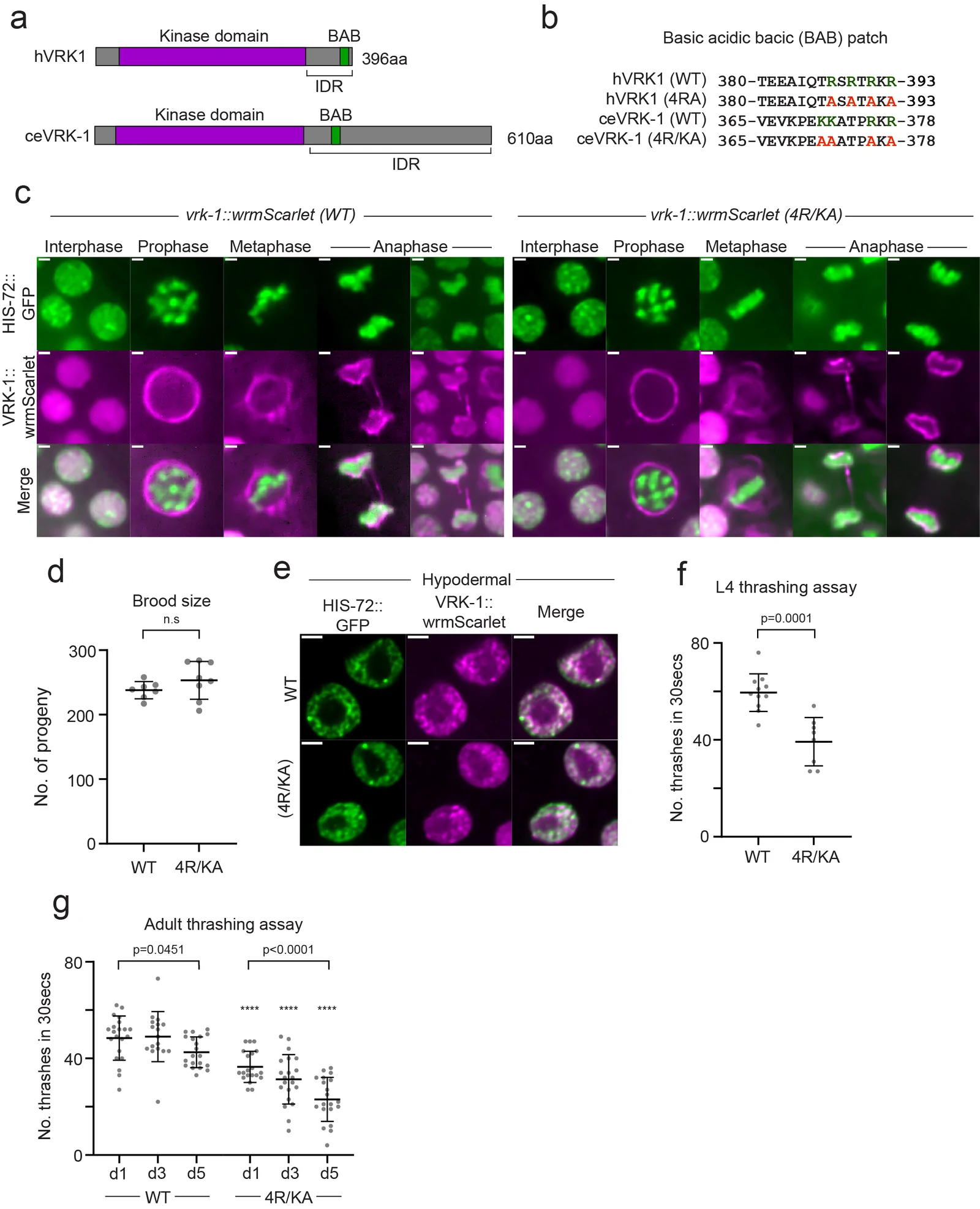



## Description

VRK1 is a serine/threonine chromatin-binding kinase with roles in a range of biological processes, including mitosis and cell cycle progression, the DNA damage response and chromatin remodeling (Lazo, 2024). VRK1 is predominantly localized in the nucleus and bound to chromatin, although a small fraction is cytosolic (Valbuena et al., 2007). The protein comprises an N-terminal kinase domain and a C-terminal disordered region that can fold over the kinase domain, adopting several conformations that modulate kinase activity and protein-protein interactions (Shin et al., 2011). In *C. elegans* , the disordered C-terminal region is considerably larger than mammalian VRK1 (Fig. 1a). Rare coding variants of VRK1 are associated with distal neuropathies in humans, a heterogeneous group of progressive diseases that primarily affect motor neurons (Lazo and Morejón-García, 2023). In model organisms, null mutants in or RNAi knockdown of VRK1 (or its orthologues) result in microcephaly, loss of neuroblast identity, reduced proliferation and motor defects (Carrasco Apolinario et al., 2023; Vinograd-Byk et al., 2018; Yakulov et al., 2014). Because of the essential role of VRK1 in mitosis, however, it is difficult to attribute these phenotypes to mitotic defects rather than other, non-mitotic pathways regulated by VRK1. Here, we show that mutations in the C-terminal region of *C. elegans* VRK-1 impair animal motor function without causing detectable mitotic defects.

We previously reported that expression of catalytically inactive VRK-1(K169E) (VRK-1 ^KD^ for kinase dead) dramatically alters chromatin architecture in hypodermal nuclei, where chromatin becomes highly enriched at the nuclear periphery - a phenotype suppressed by depletion of BAF-1, a key nuclear envelope substrate of VRK-1 (Smith et al., 2026 bioRxiv). Several other reports suggest the VRK-1/BAF-1 signaling axis is critical for detaching chromosomes from the nuclear periphery during mitosis and meiosis (Lancaster et al., 2007; Paouneskou et al., 2025; Torras-Llort et al., 2026). hVRK1 has additional chromatin substrates beyond BAF, including histone-modifying enzymes as well as histones H3 and H2A themselves (Lazo, 2024). hVRK1 has been shown to bind the negatively charged nucleosome acidic patch through its extreme C-terminal basic-acidic-basic (BAB) patch (Budziszewski et al., 2022). Mutants in which four conserved arginine residues of the BAB are substituted with alanine (hVRK1 ^4RA^ ) show abolished binding of VRK1 to nucleosomes *in vitro* and loss of association of VRK1 with mitotic chromosomes *in vivo* , based on fluorescent microscopy. *In vitro* kinase assays show that H3 threonine 3 phosphorylation is reduced by approximately 50% in VRK1 ^4RA^ mutants, whereas BAF phosphorylation is unaffected (Budziszewski et al., 2022). Because these substitutions abolish nucleosome binding while leaving BAF phosphorylation intact *in vitro* , they offer a way to probe the contribution of VRK-1 to chromatin architecture independently of the BAF-1 signaling axis. We therefore used CRISPR genome editing to introduce the equivalent VRK14 ^RA^ amino acid substitutions into the endogenously tagged VRK-1::wrmScarlet in *C. elegans* : K371A, K372A, R376A and R378A (VRK-1 ^4R/KA^ here on) (Fig. 1b). We note that these substitutions were designed to abolish nucleosome binding rather than to mimic a specific patient variant: mutation of the first arginine of the human BAB patch (R387H) is linked to distal hereditary motor neuropathy, whereas the remaining three residues have not been associated with disease.

To assess whether the 4R/KA substitutions affect chromatin binding, we compared the localization of the mutant VRK-1 with that of GFP-tagged histone H3 (HIS-72::GFP), used to visualise chromatin, and first examined mitosis and VRK-1 localization in rapidly dividing embryonic blastomeres. WT VRK-1::wrmScarlet associates with the nuclear envelope during prophase and early metaphase. Later in metaphase, WT VRK-1::wrmScarlet no longer strongly associates with chromatin, but reassociates during anaphase, often becoming enriched around mitotic chromosomes as anaphase progresses to form a shell-like structure (Fig. 1c). These observations are consistent with previous immunofluorescence studies of VRK-1 localization during mitosis in *C. elegans* , indicating that VRK-1::wrmScarlet recapitulates the localization of the endogenous protein (Gorjánácz et al., 2007). Interestingly, we found that VRK-1::wrmScarlet forms bridges between anaphase chromosomes, which have not been detected previously using immunofluorescence (Gorjánácz et al., 2007; Moura et al., 2018). In the two-colour images in Fig. 1c, HIS-72::GFP was not detectable above the local background along these VRK-1-positive strands in either VRK-1::wrmScarlet or VRK-14R/KA::wrmScarlet embryos, with histone signal there below approximately 10% and 2%, respectively, of that over the adjacent chromosome masses, whereas VRK-1::wrmScarlet along the same pixels reached roughly one third to two thirds of its own level over chromosome masses. As these are single optical sections of denoised images, we cannot exclude a histone-containing structure below our detection limit; however, our quantifications suggest that the strands are largely histone-negative. Histone-depleted connections between separating chromosomes have been described as ultrafine DNA bridges in other systems, and we note this as one possible interpretation of the structures we report. To better resolve the dynamics of VRK-1 during mitosis, we generated an endogenous VRK-1::eGFP fusion, which is substantially more photostable than VRK-1::wrmScarlet and allowed us to image VRK-1::eGFP localization over time during mitosis. At 1.6 s temporal resolution, VRK-1::eGFP showed the same overall distribution described above, and resolved changes in localization that are not apparent in single snapshots (extended data videos 1-6). During prophase, VRK-1::eGFP redistributes from the nucleoplasm to the nuclear periphery over approximately one minute, and transient VRK-1::eGFP foci appear and disappear at the nuclear periphery between consecutive frames (extended data videos 1/2). On entry into metaphase, the peripheral signal weakens and becomes discontinuous, and VRK-1::eGFP-positive structures are transiently detected across the nuclear space (extended data videos 3/4). During anaphase, a VRK-1::eGFP-positive strand spans the separating chromosome masses (extended data videos 5/6). In both timelapses this strand was present over several consecutive frames, on the order of 40 to 60 s, thinning and changing shape as the masses moved apart, and was no longer detectable while the masses continued to separate. Because these timelapses were acquired in a single channel, we cannot assign these events relative to anaphase onset or to the anaphase-to-telophase transition, and we therefore describe them relative to chromosome separation only. Establishing it would require additional markers, which we have not examined here. Acute depletion of VRK-1 in the germline results in ectopic chromosome bridges in oocytes (Paouneskou et al., 2025), and we observed a similar effect in hypodermal nuclei in nematodes expressing VRK-1 ^KD^ . We therefore suggest that anaphase VRK-1 bridges have biological relevance, at least in *C. elegans* , and are unlikely artifacts of live-imaging or tagging. Our preliminary observations of VRK-1-positive bridge-like connections between chromosome masses, in animals that show no embryonic lethality, are provocative and will require further investigation to document their frequency and to understand their nature and significance.

In contrast to hVRK1 ^4RA^ and to our surprise, the localization of ceVRK-1 ^4R/KA^ ::wrmScarlet during mitosis was largely comparable to that of WT from prophase to anaphase, and mitotic chromosomes appeared morphologically similar (Fig. 1c). Because WT VRK-1::eGFP localization changed rapidly between frames (extended data videos 1-6), and because VRK-1::wrmScarlet photobleached rapidly, we were unable to perform a quantitative analysis and therefore cannot rule out subtle defects in localization or morphology. As mitotic defects associated with VRK-1 null mutants cause sterility and embryonic lethality (Gorjánácz et al., 2007; Waters et al., 2010), we performed a brood size assay to test for such defects in *vrk-1 ^4R/KA^* , additionally scoring for embryonic lethality. We found no significant differences in brood size between VRK-1 WT and VRK-1 ^4R/KA^ (Fig. 1d), and no evidence of dead embryos, suggesting that embryonic development and germline function are broadly unimpaired in these mutants.

We next turned to somatic nuclei. We reasoned that if chromatin binding of VRK-1 ^4R/KA^ were severely impaired, its subnuclear distribution would appear diffuse. As in mitosis, VRK-1 ^4R/KA^ ::wrmScarlet localization was comparable to WT VRK-1::wrmScarlet in hypodermal nuclei, with a broad, speckle-like distribution across the nucleus that overlapped with chromatin marked by HIS-72::GFP (Fig. 1e). The overall chromatin distribution was unaffected (Fig. 1e). Finally, the morphology of VRK-1 ^4R/KA^ mutant animals was superficially wild type, in contrast to VRK-1 null mutants, which display a highly penetrant protruding vulva phenotype, most likely due to mitotic defects (Dobrzynska and Askjaer, 2016; Klerkx et al., 2009). Taken together, we conclude that the VRK-1 ^4R/KA^ mutations have no major effects on VRK-1 localization, mitosis, or broad chromatin distribution in somatic nuclei in *C. elegans* . The difference in chromatin binding between hVRK1 ^4RA^ and ceVRK-1 ^4R/KA^ could arise because *C. elegans* VRK-1 has a substantially longer disordered C-terminal region than hVRK1, raising the possibility that nucleosome binding is mediated by multiple regions of the protein. Alternatively, VRK-1 recruitment to chromatin could be mediated by cofactors absent in mammalian systems. Because we limited our study largely to VRK-1 localisation experiments, we cannot rule out other aspects such as kinase activity or protein turnover are affected in this mutant and a more detailed examination will be required in the future. For example, histone phosphorylation at H3T3 or H3S10 could still be affected in these mutants even if overall VRK-1 localization appears unchanged.

Visual inspection of animal locomotion suggested that *vrk-1 ^4R/KA^* animals appeared lethargic. We therefore performed thrashing assays to quantify locomotion behavior, counting the number of thrashes *per* animal in liquid over 30 s. We found a significant reduction (34%) in VRK-1 ^4R/KA^ L4 larvae compared to WT ones (Fig. 1f), indicating impaired motor function. Because the phenotypes caused by the vast majority of disease-causing VRK1 variants become progressively more severe over time in patients (Lazo and Morejón-García, 2023), we next assessed whether this was also the case in *C. elegans* . We measured thrashing over 30 s on days 1, 3 and 5 of adulthood. Whereas control animals showed a mild decline in thrashing frequency by day 5, *vrk-1* ^4R/KA^ mutant animals showed a progressive and significant decline over time, and moved significantly less than WT ones (Fig. 1g). In addition, *vrk-1 ^4R/KA^* mutants exhibited seizure-like behaviors, coiling for several seconds every 30 s to 1 min before resuming movement (extended data videos 7-8). Notably, we never observed seizure-like behavior after VRK-1 depletion or in VRK-1 ^KD^ mutants. Of note, coding variants or altered expression of human VRK2, the VRK1 paralogue, have been implicated as a risk factor for epilepsy (Azimi et al., 2018; International League Against Epilepsy Consortium on Complex Epilepsies, 2023; 2014; Li and Yue, 2018).

Overall, these data suggest that mutations in the BAB patch of VRK-1 broadly recapitulate aspects of human disease in *C. elegans* , while having no detectable impact on mitosis. To our knowledge, this is the first analysis of mutations in the disease-associated BAB patch of VRK1 in a model organism, beyond knockdown or null mutants. Therefore, we propose that the VRK-1 ^4R/KA^ mutant will be an effective model for 1) decoupling the mitotic and non-mitotic functions of VRK-1 and 2) further understanding the etiology of VRK1-associated distal motor neuron diseases and VRK-related epilepsy. Future studies should pursue a more systematic analysis of behavior in these mutants, coupled with the examination of muscle and nervous system architectures, including motor neuron morphology and function, to identify the perturbed molecular pathways.

## Methods

***C. elegans* maintenance**

*C. elegans* were maintained at 20 ^o^ C on NGM plates using standard laboratory procedures, as described previously (Brenner, 1974). Worms were maintained for at least three generations in non-starvation conditions before performing experiments. All experiments with *vrk-1::wrmScarlet* were performed in the *his-72::gfp* background. Strains and reagents for strain generation are described in extended data tables.

**Strain generation**

CRISPR-Cas9 mutagenesis was performed using co-CRISPR, as described previously (Smith et al., 2026 bioRxiv). For VRK-1::eGFP, eGFP was amplified with either 5' or 3' VRK-1 homology using primers rPM2149/rPM2152 and PCR products were annealed at equimolar concentrations to obtain eGFP DNA molecules with single stranded homology for both 5' and 3' sides of the C-terminal insertion site. 

**Confocal microscopy**

Embryos or L3 larvae were mounted on 2% agarose pads for imaging. Mitotic embryos were imaged at approximately the 50-100 cell stage. For larvae, levamisole was added to agarose at a final concentration of 5 mM for immobilisation. Images were obtained using Nikon Ti2 Crest v3 spinning disk microscope. Representative images were denoised using NIS elements Denoise.ai software.

**Brood size assay**

L3 worms synchronized by egg lay were transferred to individual NGM plates. Every two days the mother was moved to a fresh NGM plate. After one day, plates were checked for unhatched embryos. Two to three days after the mother was transferred, the number of progeny was scored. This was repeated until mothers had stopped laying eggs. If mothers disappeared, or were found on the side of the petri dish, they were censored from the assay. Statistical analyses were performed using Students-t test in Graphpad Prism.

**Thrashing assay**

Animals were synchronized by egg lay. 500 μl of M9 buffer was added to individual wells of a 96-well plate. Individual worms at the indicated life stage were added to the wells and allowed to acclimatise for approximately 10 minutes. Worms were censored if they failed to thrash properly after this period, as this indicates damage from the transfer. The number of thrashes was counted manually over a period of 30 s, one thrash was defined as a complete left-right-left cycle. For older adults (d3 and d5), mothers were transferred to fresh plates every two days after reaching adulthood to separate them from their progeny. Statistical analyses were performed using Students-t test in Graphpad Prism between genotypes, and one-way ANOVA within the same genotype over time.

**Table:** 

		

## Reagents

**Table:** 

** Supplementary table 1 - *C. elegans* strains and genotypes used in this study **		
**Strain**	**Genotype**	**Source**
PMW1341	*vrk-1(bq28[vrk-1::wrmScarlet]) II; his-72 (uge30[gfp::his-72]) III.*	(Smith et al., 2026 bioRxiv)
PMW1380	*vrk-1(ubs126[vrk-1::eGFP]) II. *	This study
PMW1481	*vrk-1(bq28 ubs127[vrk-1(K371A) (K372A) (R376A) (R378A)::wrmScarlet]) II;* *his-72 (uge30[gfp::his-72]) III.*	This study

**Primers, CRISPR guides and restriction enzymes used in this study**

**Table:** 

**Name**	**Sequence (5'-3')**	**Usage**	**Source**
cPM105	TGATGATTCGAGCTGCTCGT	C-terminus VRK-1 CRISPR guide, for eGPF insert	This study
rPM2149	ATGAGTAAAGGAGAAGAACTTTTCACTGG	eGFP forward primer	This study
rPM2150	TCATTTGTATAGTTCATCCATGCC	eGFP reverse primer	This study
rPM2151	AAAGCCAACATTTGATGATTCGAGCTGCTCGTCAGAAGTGGGATCCGGTGGAGGCGGGTCTGGAGGCGGGGGTAGTGGCGGGGGTGGAATGAGTAAAGGAGAAGAACTTTTCACTGG	eGFP forward primer + VRK-1 5' homology	This study
rPM2152	TGTGACTCGATTGAAGATTCCAATGGTTTACACTTTCATTTGTATAGTTCATCCATGCC	eGFP reverse primer + VRK-1 3' homology	This study
cPM111	GAGAACTACTCGAAAAGCTG	CRISPR guide for 4R/KA mutations	This study
rPM2153	ATAAAGATAATGATGAAGTGGAAGTTAAACCGGAAgcagctGCTACTCCCgctAAGgcaACTACTCGAAAAGCTGTcGAAGTCAACAATGATTCAGATGACaatgaagaac	Repair template for 4R/KA mutations	This study
rPM1830	CCGTGGTGATATTGAGATTCTTGCC	4R/KA genotyping forward primer	This study
rPM1831	CTCTTTGATAATCATCGATCGTCTGCC	4R/KA genotyping reverse primer	This study
PvuII		Digests the 4R/KA PCR product twice, the WT product once	NEB

## Data Availability

Description: Extended Data Videos 1/2. VRK-1::eGFP relocalizes from the nucleoplasm to the nuclear periphery during prophase. A single nucleus followed through prophase. At the start of the movie VRK-1::eGFP fills the nucleus, with brighter patches over the chromatin and a faint peripheral rim. Over the subsequent ~1 min the nucleoplasmic and chromatin-associated signal declines while the peripheral rim intensifies, so that by the end of the movie VRK-1::eGFP is concentrated almost entirely at the nuclear periphery. 80 frames, 1.6 s interval, 126 s total. Extended Data Videos 3/4. Loss and reorganization of peripheral VRK-1::eGFP on entry into metaphase. The bright VRK-1::eGFP rim present at the start of the movie (metaphase) becomes progressively fainter and discontinuous. Internal VRK-1::eGFP-positive structures appear transiently across the nuclear space, the peripheral outline deforms and loses its circular profile, and by the end of the movie only weak, dispersed signal remains. 60 frames, 1.6 s interval, 94 s total. Extended Data Videos 5/6. VRK-1::eGFP on separating chromosome masses (anaphase), with a transient connecting structure. Two VRK-1::eGFP-positive masses separate progressively over the course of the movie. A thin VRK-1::eGFP-positive strand is transiently visible spanning the two masses while they move apart, and is no longer detectable by the end of the movie. 80/100 frames, 126/158 s total, both at 1.6 s interval. Extended Data Stills from Videos 1 to 6Contact sheets of the six VRK-1::eGFP timelapse movies, videos 1–6 top to bottom (labelled on the left side of the images). Each block of twelve panels is drawn from one movie by sampling twelve frame indices spaced evenly across the full acquired sequence, including the first and last frames; panels read left to right, top to bottom. Mitotic stage is labelled on the left side of the images. Sampling interval and elapsed time per block: videos 1 and 5, every 7th frame of 80 (0–126 s); video 2, every 7th–8th frame of 81 (0–128 s); videos 3 and 4, every 5th–6th frame of 60 (0–94 s); video 6, every 9th frame of 100 (0–158 s). All six movies were acquired at a common 1.6 s interval. Scale bar 2 µm (bottom right). Extended Data Stills from Videos 1 to 6 (all frames)Contact sheets of the six VRK-1::eGFP timelapse movies showing all frames (panels read left to right, top to bottom). Extended Data Videos 7/8 (nematode behaviour) Extended Data Video 7 Wild-type vrk-1::wrmScarlet animals. [Bright-field] movies of three 1-day old young adult vrk-1::wrmScarlet animals in M9 medium. Movies were recorded at 0.625 frames/s and play at about 14 frames/s. Representative videos of 3 animals out of 21. Extended Data Video 8 vrk-1::wrmScarlet (4R/KA) mutants show seizure-like behavior. Movies of three 1-day old young adult animals carrying the 4R/KA mutation in vrk-1::wrmScarlet, recorded as in Extended Data Video 7. Mutant animals show seizure-like behavior. Representative videos of 3 animals out of 20. Resource Type: Audiovisual. DOI: https://doi.org/10.22002/9bsjd-8rd75 Description: Extended Data Videos 1. VRK-1::eGFP relocalizes from the nucleoplasm to the nuclear periphery during prophase. A single nucleus followed through prophase. At the start of the movie VRK-1::eGFP fills the nucleus, with brighter patches over the chromatin and a faint peripheral rim. Over the subsequent ~1 min the nucleoplasmic and chromatin-associated signal declines while the peripheral rim intensifies, so that by the end of the movie VRK-1::eGFP is concentrated almost entirely at the nuclear periphery. 80 frames, 1.6 s interval, 126 s total. Resource Type: Audiovisual. DOI: https://doi.org/10.22002/1tfx3-1ss82 Description: Extended Data Videos 2. VRK-1::eGFP relocalizes from the nucleoplasm to the nuclear periphery during prophase. A single nucleus followed through prophase. At the start of the movie VRK-1::eGFP fills the nucleus, with brighter patches over the chromatin and a faint peripheral rim. Over the subsequent ~1 min the nucleoplasmic and chromatin-associated signal declines while the peripheral rim intensifies, so that by the end of the movie VRK-1::eGFP is concentrated almost entirely at the nuclear periphery. 80 frames, 1.6 s interval, 126 s total. Resource Type: Audiovisual. DOI: https://doi.org/10.22002/31rsw-30915 Description: Extended Data Videos 3. Loss and reorganization of peripheral VRK-1::eGFP on entry into metaphase. The bright VRK-1::eGFP rim present at the start of the movie (metaphase) becomes progressively fainter and discontinuous. Internal VRK-1::eGFP-positive structures appear transiently across the nuclear space, the peripheral outline deforms and loses its circular profile, and by the end of the movie only weak, dispersed signal remains. 60 frames, 1.6 s interval, 94 s total. Resource Type: Audiovisual. DOI: https://doi.org/10.22002/yg5kz-57b30 Description: Extended Data Videos 4. Loss and reorganization of peripheral VRK-1::eGFP on entry into metaphase. The bright VRK-1::eGFP rim present at the start of the movie (metaphase) becomes progressively fainter and discontinuous. Internal VRK-1::eGFP-positive structures appear transiently across the nuclear space, the peripheral outline deforms and loses its circular profile, and by the end of the movie only weak, dispersed signal remains. 60 frames, 1.6 s interval, 94 s total. Resource Type: Audiovisual. DOI: https://doi.org/10.22002/vxbp3-7m813 Description: Extended Data Videos 5. VRK-1::eGFP on separating chromosome masses (anaphase), with a transient connecting structure. Two VRK-1::eGFP-positive masses separate progressively over the course of the movie. A thin VRK-1::eGFP-positive strand is transiently visible spanning the two masses while they move apart, and is no longer detectable by the end of the movie. 80 frames, 126 s total, both at 1.6 s interval. Resource Type: Audiovisual. DOI: https://doi.org/10.22002/36kxd-p4605 Description: Extended Data Videos 5/6. VRK-1::eGFP on separating chromosome masses (anaphase), with a transient connecting structure. Two VRK-1::eGFP-positive masses separate progressively over the course of the movie. A thin VRK-1::eGFP-positive strand is transiently visible spanning the two masses while they move apart, and is no longer detectable by the end of the movie. 100 frames, 158 s total, both at 1.6 s interval. Resource Type: Audiovisual. DOI: https://doi.org/10.22002/93p2j-02w17 Description: Extended Data Video 7 Wild-type vrk-1::wrmScarlet animals. [Bright-field] movies of three 1-day old young adult vrk-1::wrmScarlet animals in M9 medium. Movies were recorded at 0.625 frames/s and play at about 14 frames/s. Representative videos of 3 animals out of 21. Resource Type: Audiovisual. DOI: https://doi.org/10.22002/ft1x6-aet23 Description: Extended Data Video 8 vrk-1::wrmScarlet (4R/KA) mutants show seizure-like behavior. Movies of three 1-day old young adult animals carrying the 4R/KA mutation in vrk-1::wrmScarlet, recorded as in Extended Data Video 7. Mutant animals show seizure-like behavior. Representative videos of 3 animals out of 20. Resource Type: Audiovisual. DOI: https://doi.org/10.22002/985j1-jnd18
